# MEG3 Alleviated LPS-Induced Intestinal Injury in Sepsis by Modulating miR-129-5p and Surfactant Protein D

**DOI:** 10.1155/2020/8232734

**Published:** 2020-01-22

**Authors:** Xianjin Du, Dan Tian, Jie Wei, Chen Yan, Peng Hu, Xu Wu, Wenbin Yang

**Affiliations:** Department of Emergency, Renmin Hospital of Wuhan University, Hubei 430060, China

## Abstract

Sepsis and intestinal injury triggered by sepsis are common in intensive care units, which can contribute to a high mortality. lncRNAs can modulate gene expression, and they are closely involved in multiple diseases, including sepsis. In our present study, we investigated the biological function of MEG3 in sepsis, especially during the intestinal injury. Currently, we observed that in LPS-induced sepsis mouse models, the intestinal injury was triggered. Meanwhile, we reported that MEG3 was greatly decreased in vivo, with an increase of miR-129-5p and inhibition of SP-D. Then, MEG3 was overexpressed, and we found that its overexpression repressed the intestinal injury via downregulating miR-129-5p in sepsis mice. Moreover, TNF-*α* and IL-6 expression was elevated in intestinal tissues compared to the control groups. MEG3 restrained the activation of TNF-*α* and IL-6, in sepsis models. Subsequently, to induce the inflammatory injury of sepsis, human colorectal Caco2 cells were treated with 10 ng/ml LPS. 10 ng/ml LPS significantly inhibited Caco2 cell proliferation and increased the apoptosis. Additionally, MEG3 was decreased whereas miR-129-5p was obviously increased in Caco2 cells incubated with LPS. Interestingly, we showed that MEG3 repressed cell apoptosis partly and enhanced Caco2 cell proliferation. miR-129-5p overexpression could reverse the effect of MEG3 in vitro. Previously, we proved SP-D was reduced in sepsis and it depressed the intestinal injury in vivo. Finally, the correlation among MEG3, miR-129-5p, and SP-D was predicted and confirmed in our investigation. These findings indicated that MEG3 might be a potential target for intestinal damage caused by sepsis via regulating miR-129-5p and SP-D.

## 1. Introduction

Sepsis is a systemic inflammation response syndrome, which is resulted from infection. It is life-threatening, which can cause great damage to the tissues and organs [1]. In addition, sepsis is commonly resulted from the various degrees of infection after surgery, burns, or shock [2]. Currently, its incidence is about 0.3% and its mortality rate is about 20-40% [3]. Despite that great advances have been made in its management, the pathophysiology of sepsis still remains unclear.

lncRNAs are defined as a novel kind of transcripts with over 200 nts and without protein-coding capacity [4]. They can modulate genes transcriptionally or posttranscriptionally [5]. Great efforts are made to investigate the function and mechanism of lncRNAs in diseases [6–8]. Moreover, many lncRNAs have been shown to be implicated in the progression of sepsis [9]. H19 can act as a ceRNA to regulate miR-874 in LPS-induced sepsis [10]. GAS5 can promote podocyte injury in sepsis via repressing PTEN expression [11]. MEG3 is an imprinted lncRNA, which is located at chromosome 14q32 [12]. MEG3 exerts an antitumor activity in several cancers [13]. Nevertheless, the molecular roles of MEG3 in sepsis remain further illustrated.

Up to now, many investigations have reported the interplay between lncRNAs and microRNAs [14]. One famous hypothesis indicates that lncRNAs could serve as ceRNAs to segregate miRNAs from their target mRNAs [15, 16]. lncRNAs could control target mRNA expression by combining with miRNAs competitively. Whether MEG3 could function as a ceRNA to regulate sepsis progression is barely known.

Currently, we investigated the role of MEG3/miR-129-5p/SP-D in sepsis. We hypothesized MEG3 was involved in LPS-induced intestinal injury in sepsis by modulating miR-129-5p and SP-D.

## 2. Materials and Methods

### 2.1. Animal Models

This animal study was under the guidelines of the NIH for the Care and Use of Laboratory Animals and based on the guidelines of the International Association for the Study of Pain. C57/BL male mice were purchased from HFK Bioscience (Beijing, China) and kept in a room, which was temperature-controlled with a 12-hour light-dark schedule. Two groups were established randomly. 20 mg/kg LPS was used to set the sepsis models. For another, the control groups were injected with a normal saline. Vectors encoding MEG3 (LV-MEG3) and an empty lentiviral vector (LV-NC) delivering approximately 2 × 107 transforming units of recombinant lentivirus were injected into the mice of the LPS group once through the tail vein (*N* = 8 in each group). The mice were classified into 4 groups on the basis of drugs and lentivirus: the control group (without treatment), LPS group, LPS+LV-NC group, and LPS+LV-MEG3 group (*N* = 8 in each group).

### 2.2. Cell Culture

Caco2 cells were purchased from ATCC (Manassas, VA, USA) and seeded in DMEM (Solarbio, Beijing, China) with 25 mM HEPES (Solarbio, Beijing, China), 15% FBS (Sijiqing, Hangzhou, China), 100 U/ml penicillin, 100 mg/ml streptomycin, and 0.25 mg/ml amphotericin B (Sigma, St. Louis, MO, USA). LPS (Solarbio, Beijing, China) was used to treat Caco2 cells. Cells were cultured at 37°C in 5% CO2 atmosphere.

### 2.3. Transfection

Cells were transfected with miR-129-5p mimics and the NCs (GeneChem, Shanghai, China) using a Lipofectamine 2000 reagent (Invitrogen, Carlsbad, CA, USA). A recombinant lentiviral vector carrying lncR-MEG3 (Invitrogen, Carlsbad, CA, USA) was constructed.

### 2.4. QRT-PCR

Total RNA was extracted using the TRIzol reagent (Invitrogen, Carlsbad, CA, USA). Afterwards, RNAs were reversed and transcribed into cDNAs by the RT-PCR kit (Takara, Tokyo, Japan). SYBR Premix Ex Taq (Takara, Tokyo, Japan) was employed to detect MEG3 and miR-129-5p. Primers for MEG3 and miR-129-5p were obtained from GeneCopoeia (San Diego, CA, USA) and are listed in Table 1. Fold change was calculated by the equation 2 − ΔΔCt. Endogenous reference gene for MEG3 and SP-D was GAPDH. U6 was the reference gene used for miR-129-5p.

### 2.5. Cell-Counting Kit-8 Assay

CCK-8 (Dojindo Laboratories, Tokyo, Japan) was used to examine cell proliferation. Firstly, transfected cells were seeded onto 96-well plates for a whole night. Then, the CCK-8 solution (Dojindo, Kumamoto, Japan) was used to treat the cells for 2 hours at 37°C. Subsequently, the absorbance was tested at 450 nm by the multifunctional microplate reader SpectraMax M5 (Molecular Devices, Sunnyvale, CA, USA).

### 2.6. Cell Apoptosis by Flow Cytometry

Caco2 cells (5 × 105) were seeded in 6-well plates, and after transfection, cell apoptosis was analyzed using the Annexin V-FITC/PI apoptosis detection kit (BD Biosciences, San Jose, CA, USA). 1 × 105 cells were washed twice using PBS and stained by Annexin V-FITC and PI under the manufacturer's protocol. 100 *μ*l 1 × Annexin buffer was used to suspend cells, and Annexin V-FITC was used to label the cells. 5 *μ*l Annexin V and 1 *μ*l 100 *μ*g/ml PI working solution were added to cell suspension. Afterwards, the cells were incubated for about 15 minutes without light. Before cell apoptosis analysis, 400 *μ*l 1 × buffer was utilized. Apoptotic cells were distinguished using FACS can (Beckman Coulter, Fullerton, CA). Data were quantified by FlowJo software (Tree Star Inc. Ashland, OR).

### 2.7. Western Blot Analysis

Cells were washed and lysed using a M-PER protein extraction reagent. Protease inhibitors were used to incubate the lysates. Cell lysate was centrifuged for 15 minutes at 12,000 × g. Proteins were separated by 10% SDS-PAGE and transferred to PVDF membranes. Membranes were blocked in 5% skim milk for 1 hour and probed with the primary antibodies overnight at 4°C: cleaved caspase-3 antibody, SP-D antibody (Abcam, Cambridge, UK), and GAPDH antibody (Abcam, Cambridge, UK). Horseradish peroxidase-conjugated secondary antibodies were incubated with the membranes at room temperature for 1 hour. The ECL chemiluminescent kit (EMD Millipore, Billerica, MA, USA) was utilized to detect the protein bands.

### 2.8. ELISA

Supernatant of the culture medium was harvested from 24-well plates after relevant treatment. The concentrations of IL-6 and TNF-*α* were tested using the ELISA kit (R&D Systems, Abingdon, UK) in cell lysates. 100 *μ*g protein was loaded into all wells. The optical density at 450 nm was tested using the multifunctional microplate reader SpectraMax M5 (Molecular Devices, Sunnyvale, CA, USA) within 15 minutes.

### 2.9. Luciferase Activity Assay

Human MEG3 3′UTR (accession number NR_003633.3 for MEG3) and mutant MEG3 3′UTR were designed. The 3′-UTR of MEG3 gene, which contained putative targeting sites of miR-129-5p, was amplified and then inserted into pGL4.74 (GenScript, Nanjing, China). Cells were cotransfected with 0.2 *μ*g MEG3-WT or MEG3-MUT, together with 20 nM miR-129-5p mimics for 24 hours. Human SP-D 3′UTR (accession number NM_003019.5 for SP-D) and mutant SP-D 3′UTR were constructed. The 3′-UTR of SP-D gene contained putative targeting sites of miR-129-5p and was amplified and inserted into the luciferase reporter vector pGL4.74 similarly. Cells were cotransfected with 0.1 *μ*g SP-D-WT or SP-D-MUT, together with 20 nM miR-129-5p mimics. Luciferase activities were assessed at 48 hours after transfection using the Dual-Glo luciferase assay system (Promega, Madison, WI, USA) based on the manufacturer's protocol. Firefly luciferase activities were normalized to Renilla luciferase activities.

### 2.10. TUNEL Assay

Apoptotic cells of intestinal epithelium were examined using the TUNEL assay. The DeadEnd^TM^ Fluorometric TUNEL system (Promega, Madison, WI, USA) was used.

### 2.11. Immunohistochemistry Analysis

Formalin-fixed paraffin-embedded tissues were cut into 5 *μ*m paraffin sections. Antigen retrieval was conducted in heated 10 mM citrate buffer for 10 minutes. Afterwards, slides were incubated with the primary antibodies against SP-D (Abcam, Cambridge, UK). HRP-conjugated secondary antibody was used for 1 hour. Subsequently, photomicrographs were observed using the Nikon Eclipse TE 2000-Umicroscope (Nikon, Melville, NY).

### 2.12. EdU Assay

To perform the cell proliferation, EdU proliferation assay (RiboBio, Nanjing, China) was utilized. Caco2 cells were treated with 50 *μ*M EdU for 2 hours. After the incubation, cells were fixed and stained by EdU. A Leica DMI6000 B inverted microscope was used to take the images.

### 2.13. Statistical Analysis

Comparisons of continuous variables between groups were carried out using Student's *t*-test or one-way analysis of variance. GraphPad Prism 6.0. (GraphPad, San Diego, CA) was used. *P* values < 0.05 indicated statistically significant.

## 3. Results

### 3.1. MEG3/miR-129-5p/SP-D Was Involved in Sepsis Induced Intestinal Injury

**Figure 1:**
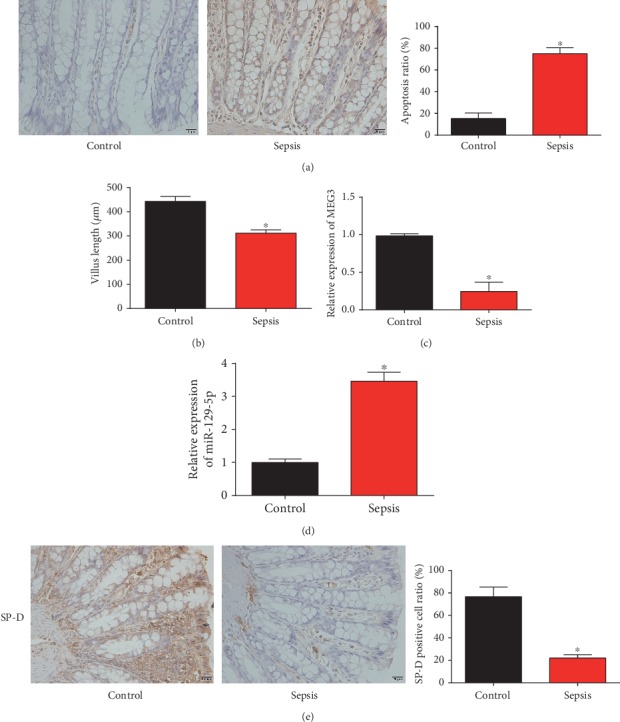
Expression of MEG3, miR-129-5p, and SP-D in LPS-induced sepsis mouse models. Intestinal tissues were obtained from the control and sepsis groups at 12 h after injection of LPS. (a) Apoptotic cells were indicated by TUNEL assay (200× magnification). Cells with brown nucleus were considered to be TUNEL-positive. (b) Villus length was quantified in sections of the jejunum. (c) MEG3 expression in the intestinal tissues. (d) miR-129-5p expression in the intestinal tissues. (e) SP-D expression in the intestinal tissues was indicated using IHC staining (200× magnification). Three independent experiments were carried out. Error bars stand for the mean ± SD of at least triplicate experiments. ^∗^*P* < 0.05.

Firstly, mice were injected using 20 mg/kg LPS to trigger sepsis models. Meanwhile, the control groups were injected with the normal saline. As exhibited in Figure 1(a), in the sepsis group, an increased intestinal apoptosis was indicated using TUNEL assay. In Figure 1(b), a significant reduction in villus length was caused in the sepsis group. Furthermore, MEG3 and miR-129-5p in intestine tissues from the mice were detected. As displayed in Figures 1(c) and 1(d), we observed MEG3 was decreased and miR-129-5p was elevated in sepsis mice. SP-D expression was downregulated in the LPS-induced sepsis group as indicated by IHC staining (Figure 1(e)). These suggested MEG3/miR-129-5p/SP-D was involved in sepsis-induced intestinal injury.

### 3.2. MEG3 Inhibited Intestinal Injury and Inflammatory Injury In Vivo

**Figure 2:**
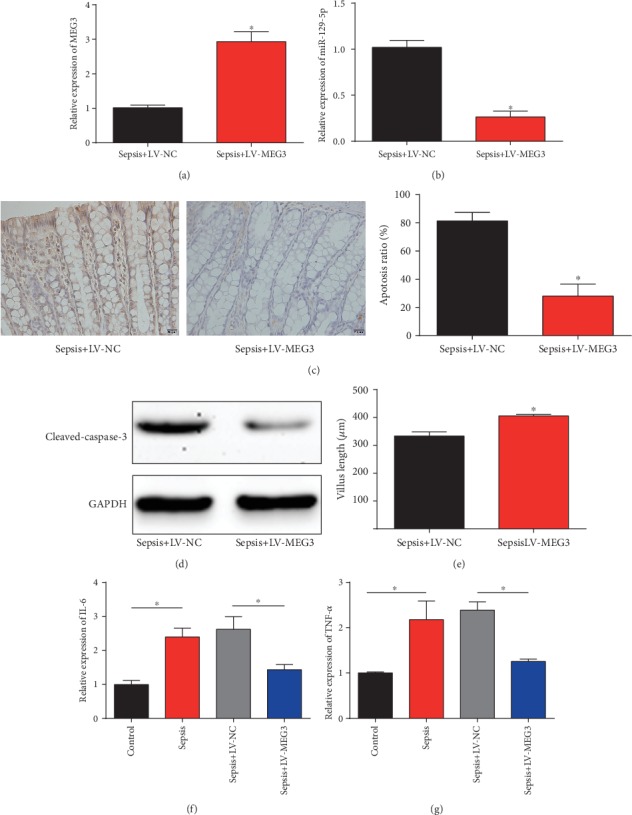
Overexpression of MEG3 inhibited the intestinal injury in vivo via downregulating miR-129-5p. (a) MEG3 expression in the intestinal tissues of the sepsis group infected with LV-NC or LV-MEG3. (b) miR-129-5p expression in the intestinal tissues of the sepsis group infected with LV-NC or LV-MEG3. (c) Apoptotic cells were indicated by TUNEL assay (200× magnification). (d) Cleaved caspase-3 protein expression was evaluated using the Western blot analysis. (e) Villus length was quantified in sections of the jejunum. ELISA analysis of IL-6 (f) and TNF-*α* (g) levels in intestine tissues. Three independent experiments were carried out. Error bars stand for the mean ± SD of at least triplicate experiments. ^∗^*P* < 0.05.

Then, to assess the effects of MEG3 on intestinal mucosal injury, MEG3 was restored in sepsis mice via infecting LV-MEG3 into the mice. In Figure 2(a), MEG3 was greatly increased after LV-MEG3 was infected. Meanwhile, miR-129-5p was dramatically decreased by the overexpression of MEG3 (Figure 2(b)). As demonstrated in Figure 2(c), mice with infected with LV-MEG3 showed a decreased apoptosis in intestinal tissues using the TUNEL assay. The present study also determined the levels of apoptosis-related protein cleaved caspase-3. Upregulation of MEG3 decreased the level of cleaved caspase-3 (Figure 2(d)). The villus length of intestine was assessed, and as shown in Figure 2(e), overexpression of MEG3 rescued the decreased villus length. Moreover, our data indicated that LV-MEG3 downregulated IL-6 and TNF-*α* protein levels in intestine tissues (Figures 2(f) and 2(g)). These implied that MEG3 inhibited intestinal injury via regulating miR-129-5p in vivo.

### 3.3. Sepsis Repressed Proliferation and Induced Apoptosis In Vivo

**Figure 3:**
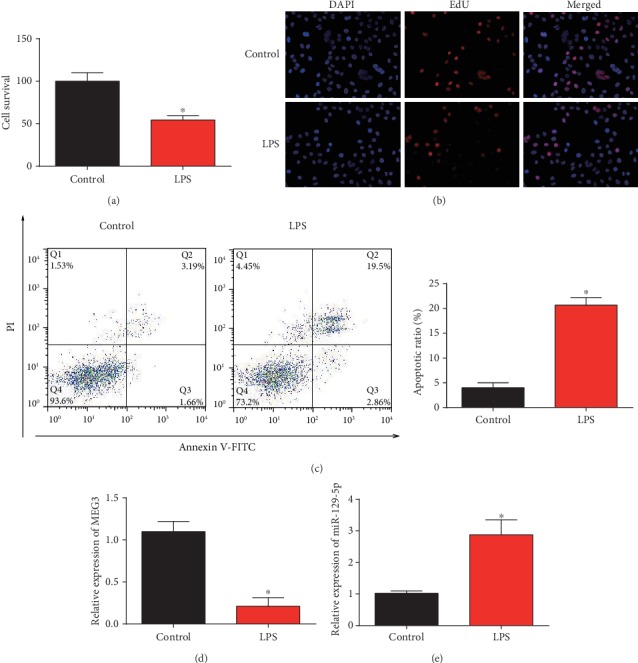
LPS-induced intestinal injury in Caco2 cells. (a) Viability of Caco2 cells after LPS treatment was measured using CCK-8 assay. (b) Proliferation of Caco2 cells after LPS treatment was tested using EdU assay. (c) Apoptosis of Caco2 cells after LPS treatment was determined using flow cytometry assay. (d) MEG3 expression in Caco2 cells after LPS treatment. (e) miR-129-5p expression in Caco2 cells after LPS treatment. Three independent experiments were carried out. Error bars stand for the mean ± SD of at least triplicate experiments. ^∗^*P* < 0.05.

Furthermore, Caco2 cells were indicated with 10 ng/ml LPS, and we found that cell viability was repressed by LPS (Figure 3(a)). EdU assay indicated Caco2 cell proliferation was repressed by LPS incubation (Figure 3(b)). In Figure 3(c), flow cytometry assay was conducted, and we found the ratio of apoptotic cells was obviously increased by LPS. The levels of MEG3 and miR-129-5p were correlated with each other negatively (Figures 3(d) and 3(e)). MEG3 was reduced while miR-129-5p was increased by 10 ng/ml LPS. These manifested that Caco2 cell proliferation was increased and cell apoptosis was induced in LPS-induced sepsis.

### 3.4. MEG3 Alleviated Intestinal Epithelial Cell Injury Triggered by LPS

**Figure 4:**
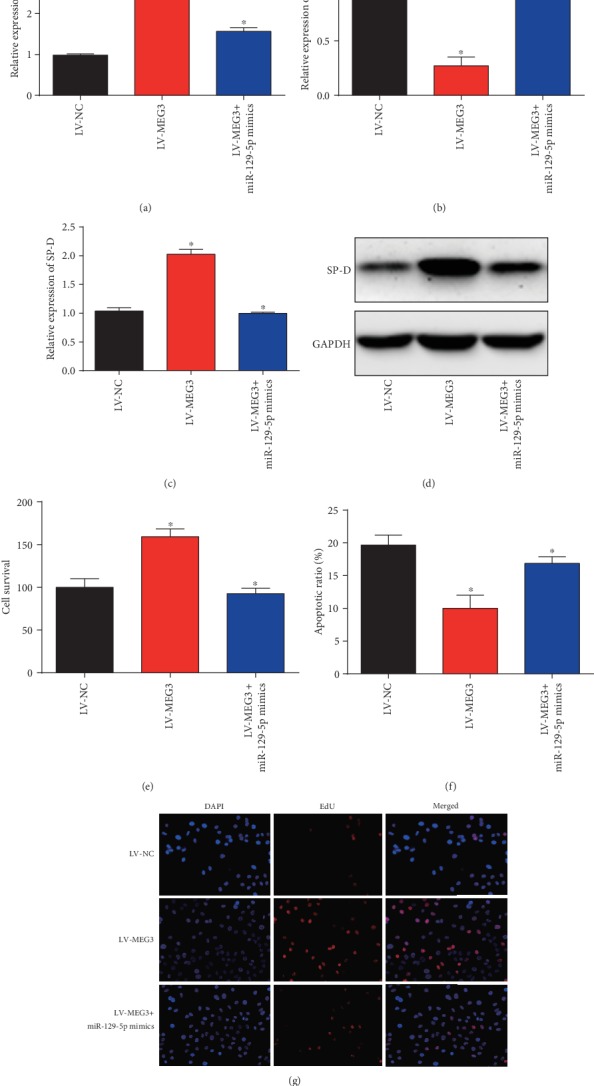
Overexpression of MEG3 alleviated LPS-induced intestinal injury in Caco2 cells. (a) MEG3 expression in Caco2 cells. Cells were infected with LV-MEG3 or LV-MEG3 and miR-129-5p mimics. (b) miR-129-5p expression in Caco2 cells. (c) SP-D mRNA expression. (d) SP-D protein expression. (e) Viability of Caco2 cells. (f) Proliferation of Caco2 cells. (g) Apoptosis of Caco2 cells. Three independent experiments were carried out. Error bars stand for the mean ± SD of at least triplicate experiments. ^∗^*P* < 0.05.

Then, Caco2 cells pretreated with LPS were infected with LV-MEG3 or LV-MEG3 and miR-129-5p mimics for 48 hours. As displayed in Figure 4(a), MEG3 was significantly increased by LV-MEG3, which could be repressed by miR-129-5p. For another, miR-129-5p was suppressed by the upregulation of MEG3 in Caco2 cells (Figure 4(b)). In Figures 4(c) and 4(d), SP-D mRNA and protein expression were induced by overexpression of MEG3, which was reduced by miR-129-5p. Then, we observed Caco2 cell survival and proliferation were obviously enhanced by LV-MEG3, which was reversed by miR-129-5p mimics (Figures 4(e) and 4(f)). Subsequently, flow cytometry analysis indicated Caco2 cell apoptosis was reduced by MEG3 while overexpression of miR-129-5p triggered the cell apoptosis (Figure 4(g)). These indicated that MEG3 improved proliferation and depressed apoptosis of Caco2 cells under LPS state.

### 3.5. miR-129-5p Was a Direct Target of MEG3

**Figure 5:**
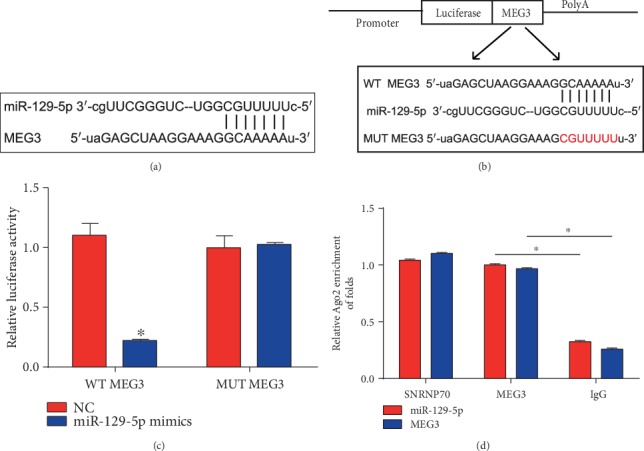
miR-129-5p served as a target of MEG3. (a) Binding sites between miR-129-5p and MEG3. (b) The luciferase reporter constructs containing the wild-type (WT-MEG3) or mutant MEG3 (MUT-MEG3) sequence. (c) WT-MEG3 or MUT-MEG3 was cotransfected into HEK-293T cells with miR-129-5p mimics or their corresponding negative controls. (d) The correlation between MEG3 and miR-129-5p was tested by RIP assay in Caco2 cells. Cellular lysates were immune-precipitated using Ago2 antibody or IgG. SNRNP70 level was employed as a positive control. Three independent experiments were carried out. Error bars stand for the mean ± SD of at least triplicate experiments. ^∗^*P* < 0.05.

Furthermore, the interaction between MEG3 and miR-129-5p was exhibited in Figure 5(a). Luciferase reporter plasmids of MEG3-WT and MEG3-MUT were displayed in Figure 5(b). Cotransfection of MEG3-WT with miR-129-5p mimics decreased the reporter activity (Figure 5(c)). In addition, we observed that MEG3 and miR-129-5p were more abundant in Ago2 pellet than in IgG pellet in Caco2 cells (Figure 5(d)). These revealed miR-129-5p functioned as a target of MEG3.

### 3.6. SP-D Was a Target of miR-129-5p

**Figure 6:**
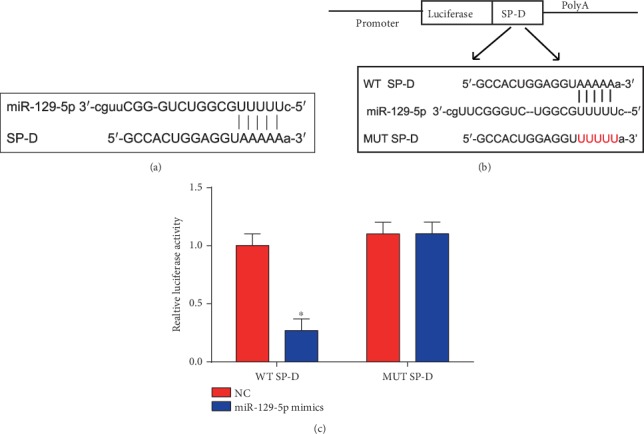
SP-D was a direct target of miR-129-5p. (a) Binding sites between SP-D and miR-129-5p. (b) The luciferase reporter constructs containing the wild-type (WT-SP-D) or mutant SP-D (MUT-SP-D) sequence. (c) WT-SP-D or MUT-SP-D was cotransfected into HEK-293T cells with miR-129-5p mimics or their corresponding negative controls. Three independent experiments were carried out. Error bars stand for the mean ± SD of at least triplicate experiments. ^∗^*P* < 0.05.

SP-D was predicted as a target of miR-129-5p in Figure 6(a). Luciferase reporter plasmids of SP-D-WT and SP-D-MUT are displayed in Figure 6(b). In addition, cotransfection of SP-D-WT with miR-129-5p mimics reduced the reporter activity (Figure 6(c)). All these observations manifested that SP-D was a target of miR-29-5p.

## 4. Discussion

Sepsis is a crucial health care issue and it has a high mortality [17]. Sepsis still remains to be a challenge due to its unsatisfactory morbidity and mortality rates [18]. Here, we found MEG3 was decreased in sepsis mouse models. Overexpression of MEG3 inhibited intestinal damage and Caco2 cell proliferation and induced cell apoptosis. miR-129-5p was elevated in sepsis, and miR-129-5p was able to reverse the effects of MEG3 in sepsis. miR-129-5p acted as a target of MEG3, and the association between them was proved. Meanwhile, SP-D was predicted and proved to be a downstream target of miR-129-5p.

The intestine can exert a crucial role in sepsis. Meanwhile, it is identified as the motor of sepsis [19]. The intestinal barrier can prevent the entry of toxins [20]. Intestinal barrier dysfunction leads to the development of sepsis [21]. Hence, we focused on the intestinal injury in sepsis progression. As exhibited, the intestinal injury was successfully induced by sepsis in vitro and in vivo.

It has been reported MEG3 can regulate IL-1*β* abundance to prevent sepsis during lung infection by acting as a decoy of miRNA [22]. Currently, we found MEG3 was decreased in sepsis mice and LPS-incubated cells. The upregulation of MEG3 disrupted the intestinal injury caused by sepsis. Increasing studies have shown proinflammatory cytokines can contribute to the intestinal barrier function [23–25]. We observed that IL-6 and TNF-*α* were induced in sepsis mouse models and MEG3 was able to restrain their protein levels.

MiRNAs regulate the gene expression through binding to the 3′-UTR of target mRNAs, which can degrade their target mRNAs and inhibit the mRNA protein translation [26, 27]. miRNAs exert important roles in many diseases, including sepsis [28, 29]. For example, miR-218 can alleviate sepsis inflammation via negatively modulating VOPP1 [30]. miR-135a aggravates the myocardial depression via regulating MAPK in sepsis [31]. In this investigation, miR-129-5p was predicted as a target for MEG3. Additionally, miR-129-5p has been shown to inhibit carcinogenesis of various tumors [32, 33]. Nevertheless, the role of miR-129-5p in sepsis has not been studied. Here, we reported miR-129-5p was upregulated in sepsis and it was negatively correlated with MEG3 expression.

Surfactant protein D (SP-D) exhibits an important role in host defense and the inflammation of various infections [34]. Previously, it has been pointed out that SP-D can inhibit the intestinal injury in sepsis [35]. Currently, we found SP-D was a target of miR-129-5p. SP-D was greatly downregulated in sepsis.

## 5. Conclusions

In conclusion, we indicated that sepsis-triggered intestinal injury might be the dysregulation of MEG3, miR-129-5p, and SP-D. These observations revealed MEG3 contributed to intestinal injury in sepsis via modulating miR-129-5p and SP-D.

## Figures and Tables

**Table 1:** Primers for real-time PCR.

Genes	Forward (5′-3′)	Reverse (5′-3′)
GAPDH	AAGAAGGTGGTGAAGCAGGC	GTCAAAGGTGGAGGAGTGGG
U6	CTCGCTTCGGCAGCACA	AACGCTTCACGAATTTGCGT
miR-129-5p	GTTGGGGAGATTTAGTTTGTT	CCTACTCCAATTCCCCCTATAATAC
MEG3	CTGCCCATCTACACCTCACG	CTCTCCGCCGTCTGCGCTAGGGGCT
SP-D	TAGATCACATGCCCACCACAT	AGCCCTTAAGCCCTGGAAGTC

## Data Availability

The data used to support the findings of this study are available from the corresponding author upon request.

## Abbreviations

CCK-8: Cell-counting kit-8
CeRNA: Competing endogenous RNA
EdU: 5-Ethynyl-2′-deoxyuridine
ELISA: Enzyme-linked immunosorbent assay
IL-6: Interleukin-6
lncRNAs: Long noncoding RNAs
LPS: Lipopolysaccharide
MEG3: Maternally expressed gene 3
PTEN: Phosphatase and tension homology deleted on chromosome ten
QRT-PCR: Quantitative real-time polymerase chain reaction
SP-D: Surfactant protein-D
TNF-*α*: Tumour necrosis factor-*α*.

## References

[1] Caserta S., Kern F., Cohen J., Drage S., Newbury S. F., Llewelyn M. J. (2016). Circulating plasma microRNAs can differentiate human sepsis and systemic inflammatory response syndrome (SIRS). *Scientific Reports*.

[2] Suresh P. K., Minal J., Rao P. S., Ballal K., Sridevi H. B., Padyana M. (2016). Volume conductivity and scatter parameters as an indicator of acute bacterial infections by the automated haematology analyser. *Journal of Clinical and Diagnostic Research: JCDR*.

[3] Moitra R., Beal D. R., Belikoff B. G., Remick D. G. (2012). Presence of preexisting antibodies mediates survival in sepsis. *Shock*.

[4] Jarroux J., Morillon A., Pinskaya M. (2017). History, Discovery, and Classification of lncRNAs. *Long Non Coding RNA Biology*.

[5] Akhade V. S., Pal D., Kanduri C. (2017). Long noncoding RNA: genome organization and mechanism of action. *Long Non Coding RNA Biology*.

[6] Bhan A., Soleimani M., Mandal S. S. (2017). Long noncoding RNA and cancer: a new paradigm. *Cancer Research*.

[7] Li X., Wu Z., Fu X., Han W. (2014). lncRNAs: Insights into their function and mechanics in underlying disorders. *Mutation Research/Reviews in Mutation Research*.

[8] Schmitz S. U., Grote P., Herrmann B. G. (2016). Mechanisms of long noncoding RNA function in development and disease. *Cellular and Molecular Life Sciences*.

[9] Sun L., Li L., Yan J. (2017). Progress in relationship of the long non-coding RNA and sepsis. *Zhonghua wei zhong bing ji jiu yi xue*.

[10] Fang Y., Hu J., Wang Z. (2018). lncRNA H19 functions as an Aquaporin 1 competitive endogenous RNA to regulate microRNA-874 expression in LPS sepsis. *Biomedicine & Pharmacotherapy*.

[11] Fang Y., Hu J. F., Wang Z. H. (2018). GAS5 promotes podocyte injury in sepsis by inhibiting PTEN expression. *European Review for Medical and Pharmacological Sciences*.

[12] Miyoshi N., Wagatsuma H., Wakana S. (2000). Identification of an imprinted gene, *Meg3/Gtl2* and its human homologue *MEG3*, first mapped on mouse distal chromosome 12 and human chromosome 14q. *Genes to Cells*.

[13] He Y., Luo Y., Liang B., Ye L., Lu G., He W. (2017). Potential applications of MEG3 in cancer diagnosis and prognosis. *Oncotarget*.

[14] Liz J., Esteller M. (2016). lncRNAs and microRNAs with a role in cancer development. *Biochimica et Biophysica Acta (BBA) - Gene Regulatory Mechanisms*.

[15] Qi X., Zhang D. H., Wu N., Xiao J. H., Wang X., Ma W. (2015). ceRNA in cancer: possible functions and clinical implications. *Journal of Medical Genetics*.

[16] Zhang Y., Xu Y., Feng L. (2016). Comprehensive characterization of lncRNA-mRNA related ceRNA network across 12 major cancers. *Oncotarget*.

[17] Singer M., Deutschman C. S., Seymour C. W. (2016). The third international consensus definitions for sepsis and septic shock (Sepsis-3). *JAMA*.

[18] Rhodes A., Evans L. E., Alhazzani W. (2017). Surviving sepsis campaign: international guidelines for management of sepsis and septic shock: 2016. *Intensive Care Medicine*.

[19] Yu Y., Yang Y., Bian Y. (2017). Hydrogen gas protects against intestinal injury in wild type but not NRF2 knockout mice with severe sepsis by regulating HO-1 and HMGB1 release. *Shock*.

[20] Lechuga S., Ivanov A. I. (2017). Disruption of the epithelial barrier during intestinal inflammation: quest for new molecules and mechanisms. *Biochimica et Biophysica Acta (BBA) - Molecular Cell Research*.

[21] Klingensmith N. J., Coopersmith C. M. (2016). The gut as the motor of multiple organ dysfunction in critical illness. *Critical Care Clinics*.

[22] Li R., Fang L., Pu Q. (2018). MEG3-4 is a miRNA decoy that regulates IL-1*β* abundance to initiate and then limit inflammation to prevent sepsis during lung infection. *Science Signaling*.

[23] Guo H., Xu Y., Huang W. (2016). Kuwanon G preserves LPS-induced disruption of gut epithelial barrier in vitro. *Molecules*.

[24] Bein A., Zilbershtein A., Golosovsky M., Davidov D., Schwartz B. (2017). LPS induces hyper-permeability of intestinal epithelial cells. *Journal of Cellular Physiology*.

[25] He L. X., Wang J. B., Sun B. (2017). Suppression of TNF-*α* and free radicals reduces systematic inflammatory and metabolic disorders: radioprotective effects of ginseng oligopeptides on intestinal barrier function and antioxidant defense. *The Journal of Nutritional Biochemistry*.

[26] Wang Q. X., Zhu Y. Q., Zhang H., Xiao J. (2015). Altered MiRNA expression in gastric cancer: a systematic review and meta-analysis. *Cellular Physiology and Biochemistry*.

[27] Lv J., Xia K., Xu P. (2014). miRNA expression patterns in chemoresistant breast cancer tissues. *Biomedicine & Pharmacotherapy*.

[28] Xie T., Huang M., Wang Y., Wang L., Chen C., Chu X. (2016). MicroRNAs as regulators, biomarkers and therapeutic targets in the drug resistance of colorectal cancer. *Cellular Physiology and Biochemistry*.

[29] Kingsley S. M. K., Bhat B. V. (2017). Role of microRNAs in sepsis. *Inflammation Research*.

[30] Li J. M., Zhang H., Zuo Y. J. (2018). MicroRNA-218 alleviates sepsis inflammation by negatively regulating VOPP1 via JAK/STAT pathway. *European Review for Medical and Pharmacological Sciences*.

[31] Zheng G., Pan M., Jin W., Jin G., Huang Y. (2017). MicroRNA-135a is up-regulated and aggravates myocardial depression in sepsis via regulating p38 MAPK/NF-*κ*B pathway. *International Immunopharmacology*.

[32] Duan L., Hao X., Liu Z., Zhang Y., Zhang G. (2014). miR-129-5p is down-regulated and involved in the growth, apoptosis and migration of medullary thyroid carcinoma cells through targeting RET. *FEBS Letters*.

[33] Xu C., Shao Y., Xia T. (2014). lncRNA-AC130710 targeting by miR-129-5p is upregulated in gastric cancer and associates with poor prognosis. *Tumor Biology*.

[34] Haagsman H. P., Hogenkamp A., van Eijk M., Veldhuizen E. J. (2008). Surfactant collectins and innate immunity. *Neonatology*.

[35] Du X., Meng Q., Sharif A. (2016). Surfactant proteins SP-A and SP-D ameliorate pneumonia severity and intestinal injury in a murine model of *Staphylococcus aureus* pneumonia. *Shock*.

